# Bayesian estimation of orientation and direction tuning captures parameter uncertainty

**DOI:** 10.3389/fncir.2025.1542332

**Published:** 2025-07-21

**Authors:** Zongting Wu, Stephen D. Van Hooser

**Affiliations:** ^1^Department of Biochemistry, Brandeis University, Waltham, MA, United States; ^2^Department of Biology, Brandeis University, Waltham, MA, United States

**Keywords:** tuning curves, motion selectivity, motion processing, spatial processing, orientation tuning

## Abstract

This study explores the efficacy of Bayesian estimation in modeling the orientation and direction selectivity of neurons in the primary visual cortex (V1). Unlike traditional methods such as least squares, Bayesian estimation adeptly handles the probabilistic nature of neuronal responses, offering robust analysis even with limited data and weak selectivity. Through the analysis of both simulated and experimental data, we demonstrate that Bayesian estimation not only accurately fits the neuronal tuning curves but also effectively captures parameter certainty or uncertainty of both strongly and weakly selective neurons. Our results affirm the complex interdependencies among response parameters and highlight the variability in neuronal behavior under varied stimulus conditions. Our findings provide guidance as to how many response samples are necessary for Bayesian parameter estimation to achieve reliable fitting, making it particularly suitable for studies with constraints on data availability.

## 1 Introduction

Neurons in the visual system of all examined mammals, as well as neurons in other systems such as head-direction cells and place cells in the hippocampus, often exhibit tuning for the orientation or direction of a stimulus (Hubel and Wiesel, 1959, 1962; Wilson et al., 2018; Hubel and Wiesel, 1968; Henry et al., 1974; Ohki et al., 2005) or of the movement of an animal in a coordinate system (Taube et al., 1990; McNaughton et al., 1983). When selectivity is strong, there are many methods for quantifying the orientation and direction angle preference, the degree of tuning relative to an orthogonal angle, or the sensitivity of the tuning as the stimulus changes from the preferred angle (Carandini and Ferster, 2000; Ringach et al., 2002; Swindale, 1998; Mazurek et al., 2014).

Today, many optophysiology and multi-channel recording studies with massive electrode channel counts often characterize the response properties of dozens or hundreds of neurons simultaneously (Stringer et al., 2019; de Vries et al., 2019; Siegle et al., 2021), some of which may have high selectivity and some of which may not. Some methods of quantifying the degree of orientation and direction tuning are robust regardless of whether tuning is strong or weak. However, reliably estimating tuning parameters such as angle preference and tuning width is not possible with least squares methods when tuning is weak (Mazurek et al., 2014). In a prior methods paper, our group recommended simply not trying to quantify parameters other than the degree or amount (or magnitude) of tuning if a statistical test (such as Hoteling's T2 test or an ANOVA across stimuli) did not show evidence of some significant tuning (Mazurek et al., 2014).

Nevertheless, there are times when one may want to quantify properties such as preference angle or tuning width even for cells that exhibit weak tuning. For example, one may want to make a statement about how tuning has changed when following a cell over the course of development, before and after an experimental manipulation, or as another parameter (say, stimulus spatial frequency, or temporal frequency) is varied (Li et al., 2008; Moore et al., 2005). If a cell is initially poorly tuned, such that we have no certainty about its angle preference, but later exhibits strong tuning for upward motion, we may want to contrast this situation with another cell that exhibits strong tuning for downward motion but later exhibits strong tuning for upward motion. If we throw up our hands when tuning is weak, we lose the ability to make quantitative statements about these situations.

Bayesian estimation methods allow quantification of a full joint distribution of the parameters of a tuning model, so that any uncertainty or certainty of a given parameter is estimated (Bishop, 2006). Full Bayesian estimation requires much more computation than least squares fitting or maximum likelihood estimation (Cronin et al., 2010), but advances in computing power and GPU methods now allow these methods to be applied routinely and at speeds that are similar to least squares approaches. We describe a Bayesian estimation approach for orientation and direction tuning curves that can be widely applied to other sensory tuning curves. We provide enough detail that an analyst who is unfamiliar with Bayesian estimation can apply the method to a new problem. We provide comparisons to the bootstrap technique (Efron and Tibshirani, 1993; Press and Flannery, 1992), which also allows one to describe the certainty or uncertainty of tuning parameters.

## 2 Materials and methods

Neurons or other systems can exhibit responses that vary with stimulus direction and/or orientation. These responses can be spikes or voltage deflections, modulation of activity at a stimulus frequency, or responses obtained from an indicator such as a calcium indictor. There are two major classes of methods for analyzing orientation and direction tuning curves: vector methods (Batschelet, 1981; Swindale, 1998; Mazurek et al., 2014) and fit methods (Carandini and Ferster, 2000; Ringach et al., 2002; Swindale, 1998; Mazurek et al., 2014). Vector methods, such as the calculation of circular variance (Ringach et al., 2002) are excellent for quantifying the degree or strength of orientation or direction tuning for weakly and strongly tuned cells (Mazurek et al., 2014), but in this paper we will focus on an equation model of orientation and direction tuning based on a wrapped Gaussian (Swindale, 1998; Batschelet, 1981; Carandini and Ferster, 2000), see Swindale (1998) for other equation choices:


(1) $$\begin{array}{l} R(\theta ;C,R_{p},R_{n},\theta_{pref},\sigma )=C+R_{p}*e^{-\frac{{\left(\text{angdiff}(\theta -\theta_{pref})\right)}^{2}}{2\sigma^{2}}}\\ \;+R_{n}*e^{-\frac{{\left(\text{angdiff}(\theta -\theta_{null})\right)}^{2}}{2\sigma^{2}}}. \end{array}$$

Five parameters are included in the equation, which are *C*, *R*_*p*_, *R*_*n*_, θ_*pref*_ and σ. The offset, *C*, is the portion of the response that is constant and does not vary with orientation or direction. *R*_*p*_ is the response above offset for the preferred direction and *R*_*n*_ is the response above offset for the null direction, that is, the direction that is opposite of the preferred direction. θ_*pref*_ is the preferred direction. In direction space, the range of θ is [0, 360°), and $\theta_{null}=\theta_{pref}+180^{•}$. The function angdiff(θ) = *min*(θ, θ−360, θ+360) computes the absolute angular difference around the circle. We also sometimes refer to the response in the preferred direction and the null direction, respectively, as


(2) $$\begin{array}{l} R_{pref}=R\left(\theta_{pref}\right) \end{array}$$

and


(3) $$\begin{array}{l} R_{null}=R\left(\theta_{null}\right). \end{array}$$

Various different groups use different definitions of θ, with some using Cartesian coordinates where 0° refers to vertical bars moving to the right, and increasing θ implies a counter-clockwise change in angle, and other groups such as own using compass coordinates, where 0° refers to horizontal bars moving upwards and increasing θ implies a clockwise change in angle.

### 2.1 Bayesian formulation

Bayes' theorem is stated mathematically as the following equation:


(4) $$\begin{array}{l} P\left(A|B\right)=\frac{P\left(B|A\right)P\left(A\right)}{P\left(B\right)}. \end{array}$$

We constructed a Bayesian model of Equation 1. We employed a change-of-variable by letting *R*_*n*_ = α*R*_*p*_ with 0 ≤ α ≤ 1, so that we can always be sure that *R*_*n*_ ≤ *R*_*p*_ as we compute the likelihood of the model. Our new equation is as follows:


(5) $$\begin{array}{l} P\left(C,R_{p},\alpha ,\theta_{pref},\sigma |D\right)=\frac{P\left(D|C,R_{p},\alpha ,\theta_{pref},\sigma \right)P\left(C,R_{p},\alpha ,\theta_{pref},\sigma \right)}{P\left(D\right)}. \end{array}$$

In words, the equation says that the probability of the parameters (*C, R*_*p*_, α, θ_*pref*_, σ) given the data *D* we observed is equal to the probability of seeing the data given the parameters, multiplied by the prior probability of the parameters and divided by the prior probability of the data (Bishop, 2006).

The left side of Equation 5 describes what we are seeking: how likely is it that each parameter (say, *C*), takes any of a set of values (say, {*C*_1_, *C*_2_, *C*_3_, …}). In order to calculate this probability, we need to be able to calculate the probability of the data given the parameters (called *the likelihood function*) and the prior probability of the parameters.

In our case, the data *D* is the set of empirical mean responses across trials to the particular angles we examined. We indicate each of *i* angles tested by θ_*i*_ and the mean response to that angle as *r*_θ*i*_. We need to calculate the probability, according to our model, that we observed *r*_θ*i*_ given the parameters. Equation 1 tells us that the expected response ${\hat{r}}_{\theta i}$ for parameters (*C, R*_*p*_, α, θ_*pref*_, σ) is


(6) $$\begin{array}{l} {\hat{r}}_{\theta i}=R\left(\theta_{i};C,R_{p},\alpha ,\theta_{pref},\sigma \right). \end{array}$$

Next, we need to take this expected response and calculate the likelihood of the actual response *r*_θ*i*_ in units of probability. Many studies of the variability of neural firing rates have found that the variance (and standard deviation) of spike responses is linearly related to the mean response rate on a log-log plot (Softky and Koch, 1993; Shadlen and Newsome, 1998). We therefore assumed that the model responses would be drawn from a normal distribution with mean ${\hat{r}}_{\theta i}$ and a standard deviation σ_*noise*_ that was a function of the mean response. To estimate σ_*noise*_(*m*) for our data, we started by examining the log of the standard deviation of each response σ_*noise, i*_ of each cell as a function of the log of the mean response *m*_*i*_:


$$log\left(\sigma_{noise,i}\right)=a+b*log\left(m_{i}\right).$$

If one were to obtain the best-fit values of *a* and *b* then one could express the expected variability as a function of the mean response as


$$\begin{array}{l} \sigma_{noise}\left(m\right)=10^{a}*m^{b}. \end{array}$$

We modified this classic function (Softky and Koch, 1993; Shadlen and Newsome, 1998) slightly so that the noise is not identically 0 at 0 mean rate, which leads to strange parameter estimations, and to achieve better fits of the noise at the lowest mean rates, arriving at a final noise equation with three fitted constants *C*, *K*, and *S*:


(7) $$\begin{array}{l} \sigma_{noise}\left(m\right)=C_{n}+K*m^{S}. \end{array}$$

For each set of recordings, we pooled our observations of all σ_*noise, i*_ and *m*_*i*_ over all responses and all cells and derived a single set of *C*_*n*_, *K*, and *S* that was applied to all cells. For example, when dealing with calcium imaging data from ferret visual cortex (Li et al., 2008), we used all cells and all responses to calculate the constants we used for the noise model for those cells. We did not derive these constants for each cell individually (as the limited data for a single cell would produce a poor fit), or apply *C*_*n*_, *K* and *S* across situations (for example, between recordings of spiking neurons and recordings using calcium indicators).

Analysts are encouraged to examine the fit of the noise on a log-log plot for each new application. While our data were well fit, and previous studies have found this function describes the relationship between mean spike rates and variability for data in cortex (Softky and Koch, 1993; Shadlen and Newsome, 1998), factors such as calcium indicator saturation (Nauhaus et al., 2012) or different firing properties in different circuits might necessitate using a modified noise function.

In our experiments, we usually record responses over several trials *T* rather than recording a single trial, and report the mean response averaged over trials *r*_θ*i*_. Because we average over trials, we expect the response we measure to be less noisy than the results of a single trial. According to the central limit theorem, the variability decreases by a factor of $1/\sqrt{\left(T\right)}$. Therefore, the probability that a response at a single stimulus angle was generated by a particular set of model parameters is


(8) $$\begin{array}{l} P\left(r_{\theta i}|C,R_{p},\alpha ,\theta_{pref},\sigma \right)=N\left(r_{\theta i};{\hat{r}}_{\theta i},\sigma_{noise}\left({\hat{r}}_{\theta i}\right)/\sqrt{\left(T\right)}\right) \end{array}$$

where *N*(*x*; μ, σ_*normal*_) is the probability density function of the normal distribution with mean μ and standard deviation σ_*normal*_. We assume that the response measurements are independent, so that the probability of seeing the set of responses {*r*_θ1_, *r*_θ2_, …, *r*_θ*n*_} for each cell is


(9) $$\begin{array}{l} P\left(D|C,R_{p},\alpha ,\theta_{pref},\sigma \right)=\overset{i=n}{\underset{i=1}{\prod }}N\left(r_{\theta i};{\hat{r}}_{\theta i},\sigma_{noise}\left({\hat{r}}_{\theta i}\right)/\sqrt{\left(T\right)}\right). \end{array}$$

Note that the expected response in the noise equation is based on the model response. In this report, we did not make any assumptions about the *a priori* likelihood of the different values of the parameters (*C, R*_*p*_, α, θ_*pref*_, σ). We estimated our distributions for discrete sets of values of each parameter (for example, C could take {*C*_1_, *C*_2_, *C*_3_, …, *C*_*CM*_}, *R*_*p*_ could take {*R*_*p*1_, *R*_*p*2_, *R*_*p*3_, …, *R*_*p*_*RPM*}, etc), and we assumed each value was equally likely so that


(10) $$\begin{array}{l} P\left(C,R_{p},\alpha ,\theta_{pref},\sigma \right)=\frac{1}{\left(CM\right)\left(RPM\right)\left(\alpha_{M}\right)\left(\theta_{M}\right)\left(\sigma_{M}\right)}, \end{array}$$

where *CM*, *RPM*, α_*M*_, θ_*M*_, and σ_*M*_ are the number of values of each parameter used to estimate the distribution. Perhaps in the future, by examining many populations, the expected prior distributions could be estimated differently from a uniform distribution, but we did not want to impose any biases at this time.

Finally, we did not explicitly compute *P*(*D*), but we assumed that our calculation of *P*(*C, R*_*p*_, α, θ_*pref*_, σ|*D*) would estimate the real probability distribution, and reported the estimated distribution normalized to 1. We end up with a matrix of likelihoods *L*(*c, r*, α_*j*_, *t, s*) with one entry for each combination of parameters examined.

### 2.2 Practical computation and storage of Bayesian results

We calculated our Bayesian distributions using ranges of parameters that varied slightly depending upon the units of the data. In our case, we used mean spike rates and fractional change in calcium indicators from baseline. The ranges we used are shown in Table 1.

**Table 1:** Parameter space sampled for Bayesian parameter estimation, for spiking neurons (left column) and calcium imaging (right column).

**Parameter**	**Spiking neurons**	**Calcium imaging**
*min*(*C*)	0.1	−*MX*
*max*(*C*)	10	+*MX*
*CM*	60	60
*min*(*R*_*pref*_)	0.1	0.001
*max*(*R*_*pref*_)	20	+3*MX
*R*_*pref*_ *M*	60	60
*min*(α)	0	0
*max*(α)	1	1
α*M*	15	21
*min*(θ_*pref*_)	0	0
*max*(θ_*pref*_)	359	359
θ_*pref*_*M*	72	72
*min*(σ)	1	1
*max*(σ)	60	60
σ*M*	60	60
	Number of calculations	Number of calculations
	2.3*10^8^	3.3*10^8^
		

Parameters were varied from a minimum value to a maximum value using the number of steps indicated for each parameter with an *M*. For example, *CM* is the number of parameters evaluated for parameter *C*. For calcium imaging, *MX* indicates the empirical maximum mean response for each cell. The total number of calculations necessary for each cell is shown at the bottom. The offset *C* is allowed to vary between −*MX* and *MX* because cells may exhibit stimulus responses that are increased or decreased relative to the nearest blank/control stimulus.

To perform Bayesian estimation for a single cell, one must perform calculations for all combinations of the 5 parameter ranges, and Table 1 indicates that billions of calculations are required for each cell. This creates burdens for computation and for storage of the results. Fortunately, modern GPUs are well-suited to performing these calculations quickly. We wrote GPU code using Matlab (MathWorks, Natick, MA) that performs these calculations in less than a minute per cell on an ordinary laptop with a GPU, so the computation is not problematic for today's computing environments.

Storing the results requires careful decisions, however. Each probability for every parameter combination is a floating point number; if stored on disk with double precision, then the results for a single cell would require on the order of 10 GB of storage. For this reason, we did not store all of the results but instead stored a much briefer summary of key statistics.

First, we stored the marginal likelihood of each parameter individually by summing over all other parameters and normalizing the distribution to 1. For example, the marginal likelihood $\hat{Lc}\left(c\right)$ of parameter *C* is computed as


$$Lc\left(c\right)=\overset{r=RPM}{\underset{r=1}{\sum }}\overset{\alpha_{j}=\alpha_{M}}{\underset{\alpha_{j}=1}{\sum }}\overset{t=\theta_{M}}{\underset{t=1}{\sum }}\overset{s=\sigma_{M}}{\underset{s=1}{\sum }}L\left(c,r,\alpha_{j},t,s\right)$$

(11) $$\begin{array}{l} \hat{Lc}\left(c\right)=\frac{Lc\left(c\right)}{\sum_{c=1}^{c=CM}Lc\left(c\right)}. \end{array}$$

While we lose information about the joint distributions of these parameters, the summary provides considerable savings, needing only (*CM*) + (*RPM*) + (α_*M*_) + (θ_*M*_) + (σ_*M*_) entries for storage instead of (*CM*)(*RPM*)(α_*M*_)(θ_*M*_)(σ_*M*_) entries (the sum instead of the product), which with our parameters means a difference between kilobytes and gigabytes per cell. The marginal distributions say a lot about the response properties of the cell, and, if we really need the joint distribution, we can recompute it.

Second, we performed estimates of the likelihoods of index values of interest while we had the full matrix of parameter estimations in memory, and stored histograms of these values. *OI* (Orientation Index) and *DI* (Direction Index) are two indexes of neuronal direction/orientation selectivity. Their magnitudes are used to characterize the sensitivity of neurons to stimuli motion in different directions/orientations in space. In this paper, we define these values as follows:


(12) $$\begin{array}{l} OI=\left(R_{pref}+R_{null}-\left(R_{orth+}+R_{orth-}\right)\right)/\left(R_{pref}+R_{null}\right) \end{array}$$

and


(13) $$\begin{array}{l} DI=\left(R_{pref}-R_{null}\right)/R_{pref}. \end{array}$$

For summary statistics, we computed histograms of the likelihood that *OI* and *DI* took values between 0 and 1 in bins of size 0.05. By using the full likelihood matrix to calculate these histograms but only storing the histogram results, we obtained a good picture of our certainty or uncertainty about the value of each index that uses the full joint information, while storing only 20 values (1/0.05) per index.

Third, we also stored the most likely value of the full joint parameters also known as the maximum likelihood estimate (MLE). These parameters are a single set of values of *C, R*_*p*_, α, θ_*pref*_, σ that provided the largest joint likelihood, but by themselves do not account for our uncertainty or certainty of their values.

### 2.3 von Mises test

To test the framework's ability to characterize the activity of tuning curves that are generated by similar but not exact matches for the model, we generated some simulated orientation tuning curves using the von Mises function (Batschelet, 1981; Swindale, 1998):


(14) $$\begin{array}{l} R_{v-m}\left(\theta \right)=Aexp\left(k\left[cos2\left(\theta -\varphi \right)-1\right]\right). \end{array}$$

### 2.4 Relative error in parameter estimation

In order to quantify the performance of the Bayesian parameter estimation, we devised a relative error measurement. Given the *true* value *x*_*t*_ of a given parameter, we defined the expected squared error (ESE) of the Bayesian parameter estimation as:


(15) $$ESE=\int_{a}^{b}(\hat{Lc}(x)*{\left(x-x_{t}\right)}^{2}dx,$$

and the expected squared error for a uniform distribution as


(16) $$\begin{array}{l} ESE_{u}=\int_{a}^{b}{\left(x-x_{t}\right)}^{2}dx. \end{array}$$

Finally, we defined the relative error of our approach compared to an uninformed (uniform) estimate as *ESE*/*ESE*_*u*_.

### 2.5 Source code and data sources

Code in MATLAB that performs the Bayesian parameter estimation in a manner callable from other projects is provided at https://github.com/VH-Lab/vhlab-bayesoridir-matlab. The repository also includes all code that was used to generate the figures in the paper.

Some data for the figure on noise were obtained from the Allen Brain Observatory (de Vries et al., 2020) using the Brain-Observatory-Toolbox in Matlab (https://github.com/MATLABCommunity-Toolboxes-at-INCF/Brain-Observatory-Toolbox).

## 3 Results

**Figure 1:**
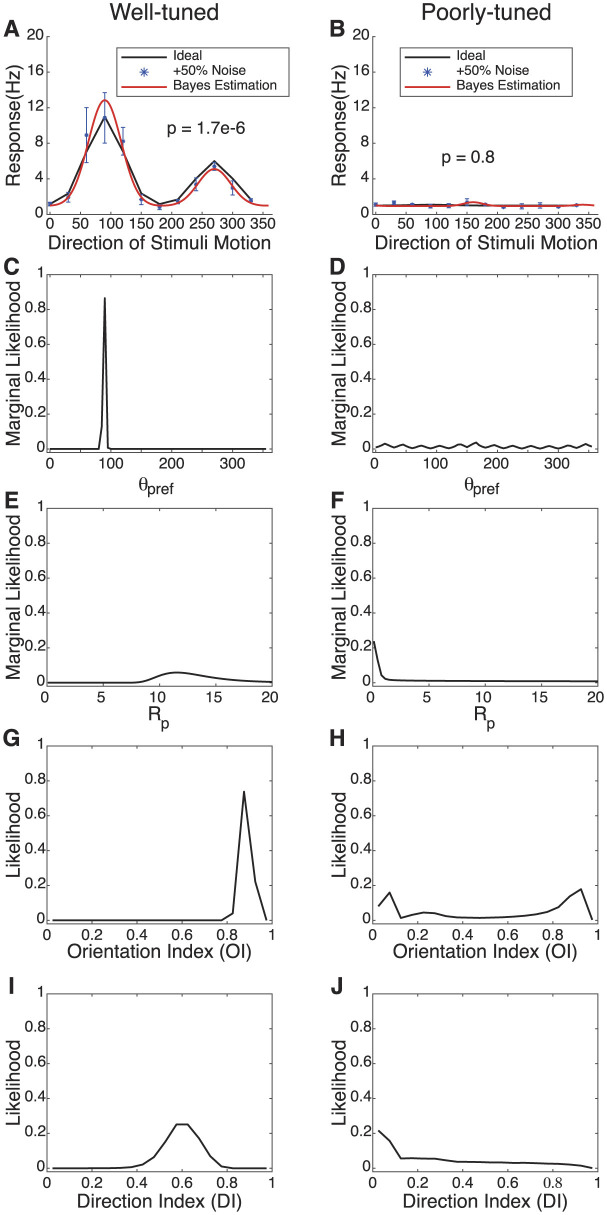
Bayesian estimation for two types of simulated cells with different levels of tuning. **(A, B)** Simulated response curve, data collection points with +50% Gaussian noise, and Bayesian estimation results. The thick black line indicates the “true” response of the neuron with no noise added, and the blue datapoints indicate simulated responses with noise added. The red line reflects the most likely parameters from the Bayesian parameter estimation. P values indicate the result of an ANOVA test across stimuli; the well-tuned cell is highly significant while the poorly-tuned cell is not. **(C–F)** The marginal likelihoods of θ_*pref*_ and *R*_*pref*_ in the model. **(G–J)** The probability distribution of *OI* and *DI* for spatial selectivity under Bayesian estimation.

To illustrate the approach and to explore how the Bayesian estimation tracks a tuning curve's parameters under a ground truth situation, we generated simulated data for analysis. In Figure 1, we show two example tuning curves: one is well-tuned for orientation and direction (Figure 1A) while the other is poorly tuned (Figure 1B). The output of Bayesian estimation is not a single value of each parameter but instead is a probability distribution over the parameters. The marginal likelihood for θ_*pref*_ and *R*_*pref*_ is plotted for each cell, along with histograms of the estimated orientation index (OI) and direction index (DI). For example, in Figure 1C, we do not obtain a single “best fit” or “most likely” value for θ_*pref*_ but a likelihood distribution, where the estimated probability that θ_*pref*_ takes any value in the range. The entire shape of the curve, not just the peak, is important to the interpretation. When the tuning is strong, there is great certainty about the parameters and the values of the index values. When the tuning is weak, there is much more uncertainty. In particular, in the weakly-tuned case, there is great uncertainty about whether the orientation and direction index values are near 0 or might be moderate. Least-squares methods would give us a single value of all of these parameters, and our uncertainty about the orientation and direction index values would not be described.

One may ask, why bother to study the tuning properties of weakly-tuned cells? To demonstrate such a case, we imagine measuring the direction tuning of a single cell while another visual stimulus parameter (temporal frequency) is changed. For low temporal frequency, the cell exhibits high direction selectivity; for a moderate temporal frequency, the cell exhibits orientation tuning but not direction tuning; for a further temporal frequency, the cell is weakly responsive.

To evaluate the predictive accuracy and sensitivity of the Bayesian estimation model, we simulated response curves for two different types of cells and performed parameter estimation. All error bars in the text are represented using standard errors of the mean (SEM).

### 3.1 Bayesian estimation for simulated strong and weak spatial selectivity

As shown in Figure 1, multiple graphs illustrate the response characteristics of simulated neurons. The left column, labeled as “Well-tuned,” features graphs that exhibit neuronal responses with higher peak firing rates and marked preferences for specific directions and orientations of stimuli. These responses are quantified in terms of frequency (Hz) and marginal likelihood across different orientations and directions, denoted by directional and orientation indexes. The right column, labeled as “Poorly-tuned,” displays neurons with significantly lower response amplitudes, less distinct tuning curves, indicating a reduced selectivity for stimulus features. As shown in Figures 1A, B, the black curves represent the ideal response curve of simulated data without any noise. To approximate real data more closely, we used simulated sampling points with an added 50% Gaussian noise for fitting. Each data point is based on five samples, covering a total of 36 angles. These sampling points are represented as blue dots in the figure. The noisy data, alongside results from a linear noise model, serve as inputs for Bayesian estimation, which are then interpolated based on given parameter ranges to produce the fits displayed as red curves. The parameter settings for the well-tuned simulated cells are (*C* = 1, *R*_*pref*_ = 10, *R*_*null*_ = 5, $\theta_{pref}=90^{•}$, and σ = 30°), while those for the poorly-tuned cells are (*C* = 1, *R*_*pref*_ = 1, *R*_*null*_ = 0, $\theta_{pref}=90^{•}$, and σ = 30°).

The Bayesian MLE fit for both types of simulated cells closely align with the ideal curves, particularly for the well-tuned cell in terms of the preferred angle and tuning width values. Figures 1C–F describe the marginal probability distributions for θ_*pref*_ and *R*_*p*_. The probability peaks accurately estimate the “true values” of θ_*pref*_ and *R*_*pref*_ for the well-tuned cell. The certainty or uncertainty of the parameters is indicated by the probability distribution peaks and widths. Due to the poorly-tuned cell exhibiting weak selectivity for direction, the estimated probability for θ_*pref*_ is dispersed across a broader range of angles compared to the well-tuned cell. The likely values of *R*_*pref*_ are considerably smaller for the cell with poorer tuning, showing its reduced response.

Figures 1G–J illustrates the probability distributions for the selectivity indexes *DI* and *OI*. It can be observed that the well-tuned cell displays distinct, singular probability peaks for DI and OI, accurately estimating the “real” values (*OI* = 0.86, *DI* = 0.45). In contrast, for the poorly-tuned cell, the likelihood estimates of both are broader, although a close estimation to the “real” value (*OI* = 0.33, *DI* = 0.5) is also observed in the probability distribution (*OI* = 0.3, *DI* = 0.38).

### 3.2 Storing marginal likelihoods as a summary

Storing the full joint likelihood distribution *P*(*C, R*_*p*_, α, θ_*pref*_, σ|*D*) requires gigabytes of storage. For many purposes, viewing or storing only the marginal likelilood of each parameter, as we have plotted in Figure 1 is sufficient. Of course, there is some information in the full joint likelihood function that might be needed for certain applications.

**Figure 2:**
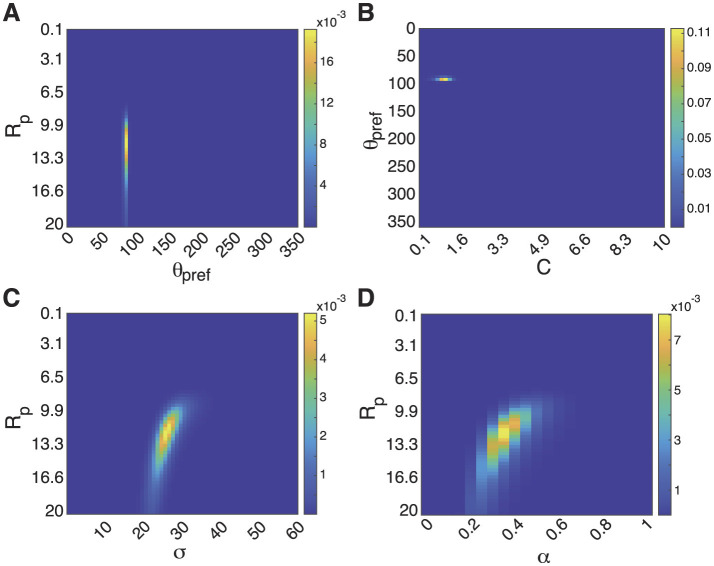
Views of joint likelihood across parameters for the strongly tuned cell in Figure 1. **(A)** Joint likelihood density plot of θ_*pref*_ and *R*_*p*_, marginalized over all other parameters. The lack of a slant indicates that these likelihoods are independent and that the individual marginal likelihoods are a good summary of the whole. Scale bar indicates probability in density plot. **(B)** Joint likelihood of θ_*pref*_ and *C*. **(C)** Joint likelihood of *R*_*p*_ and α. Slant indicates some joint dependence of these parameters. The information in the slant is lost when summarizing only the marginal likelihoods of each parameter, and might need to be stored for some applications. **(D)** Joint likelihood of *R*_*p*_ and σ.

We illustrate 2-dimensional views of the joint likelihood for pairs of parameters (marginalized over other parameter) in Figure 2. The relationships between some parameters are quite independent. For example, the joint likelihood of θ_*pref*_ and either *R*_*p*_ (Figure 2A) or *C* (Figure 2B) show little interaction among the two parameters. For some parameters, such as between *R*_*p*_ and α (Figure 2C) or σ (Figure 2D), there is evidence of some correlation. Some scientists might be interested in this fine structure for some applications, and might need to store more information or to recompute it as needed.

### 3.3 Bayesian estimation for simulated temporal frequency

Many studies of orientation or direction selectivity in the visual cortex employ drifting sinusoidal gratings (with a particular spatial frequency and temporal frequency) as stimuli (e.g., Movshon et al., 1978). Some tuning properties, such as orientation tuning, are not very sensitive to spatial or temporal frequency, but others, such as direction selectivity, can change with stimulus parameters (Moore et al., 2005). For example, Moore et al. (2005) found that many cells in ferret visual cortex altered their direction preference angle or the amount of direction selectivity as temporal frequency was changed. Direction selectivity was often higher at low temporal frequencies than at higher temporal frequencies. If one wanted to study, say, how direction preference changed with temporal frequency, one would be out of luck with least squares approaches if selectivity became low. Here we will illustrate that the Bayesian approach allows one to study these properties quantitatively.

**Figure 3:**
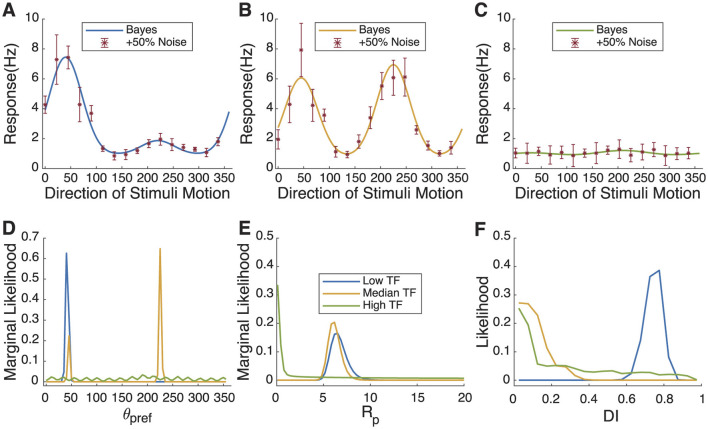
Neuronal responses and Bayesian estimation to stimuli at different temporal frequencies in simulated cell. **(A–C)** Simulated “real” response curves and sampling points with 50% added Gaussian noise in different temporal frequencies. **(D–F)** The probability distribution of θ_*pref*_, *R*_*pref*_, and *DI* fitted for different temporal frequencies. The changes in the likelihood of θ_*pref*_ and *DI* track the increased uncertainty of θ_*pref*_ with increasing temporal frequency and the decreasing *DI* with temporal frequency.

**Figure 4:**
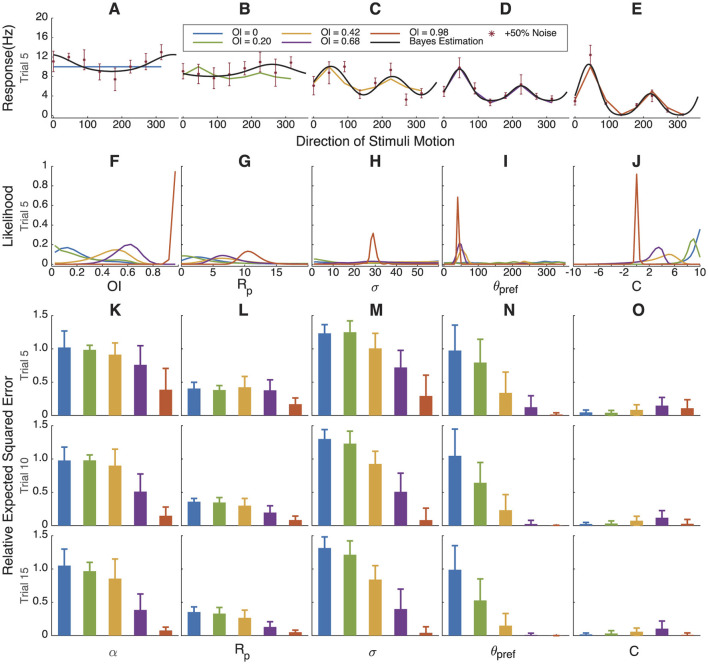
Bayesian estimation results for simulated cells with gradually increasing orientation index values. **(A–E)** Simulated response curve, sampling points with +50% Gaussian noise and fitting results. **(F–J)** The marginal likelihoods distribution of *OI*, *R*_*pref*_, σ, θ_*pref*_ and *C*. As direction selectivity progresses from high to low to non-existence, θ_*pref*_, *R*_*p*_, and *DI* track accordingly. **(K–O)**. Relative error in parameter estimation compared to assuming a uniform distribution for each parameter. When orientation selectivity is high (red), we have certainty about the values, but when orientation selectivity is low, our certainty comes close to a uniform distribution (relative error of 1). 500 simulations of 5, 10, or 15 trials.

In Figure 3, we simulated changes in direction selectivity of a single neuron under three different temporal frequencies: low, medium, and high. In our simulations, we hypothesized that the direction selectivity is strongest at low temporal frequency (*C* = 1, *R*_*pref*_ = 7, *R*_*null*_ = 1, $\theta_{pref}=45^{•}$, and σ = 30°). The maximum response amplitude is slightly smaller at the medium temporal frequency (*C* = 1, *R*_*pref*_ = 5, *R*_*null*_ = 5, $\theta_{pref}=45^{•}$, and σ = 30°), and the direction selectivity is very low. At the highest temporal frequency (*C* = 1, *R*_*pref*_ = 0, *R*_*null*_ = 0, $\theta_{pref}=45^{•}$, and σ = 30°), responses are very weak and there is an absence of direction selectivity. As shown in Figures 3A–C, red represents the simulated sampled data points, the blue curve represents the simulated ideal curve for low temporal frequency, and yellow for medium temporal frequency, and green for high temporal frequency. To more closely replicate the actual fitting process, the number of sampling angles was reduced from 32 in Figure 1 to 16. Figures 4D–F shows the changes in the probability distributions of θ_*pref*_, *R*_*pref*_, and *DI* across the three different temporal frequencies, with the colors corresponding to the three temporal frequencies mentioned above. In Figure 3D, Bayesian estimation accurately identifies the preferred angle at low temporal frequency. At the medium temporal frequency, since the response rates at *R*_*pref*_ and *R*_*null*_ are close, the model indicates two probability peaks at θ_*pref*_ and θ_*null*_. For the high temporal frequency, the probability distribution lacks distinct peaks. This is further supported in Figures 3E, F, where the probability peak of *R*_*pref*_ and *DI* at high temporal frequency predominantly cluster near or at zero, indicating minimal direction selectivity. For the medium temporal frequency, the obvious peak around 6 for *R*_*pref*_ and the peak near zero for *DI* suggest that the 'real' response curve has significant orientation selectivity with similar values of *R*_*pref*_ and *R*_*null*_. The parameters and direction index for all three temporal frequencies successfully approximate the 'true values'.

### 3.4 Bayesian estimation fitting for simulated data with variable orientation index and direction index values

To better test the universality and accuracy of Bayesian estimation, we simulated two groups of neurons, each consisting of five cells with gradually increasing *OI* or direction index *DI* values, ranging from 0 to 1.

**Figure 5:**
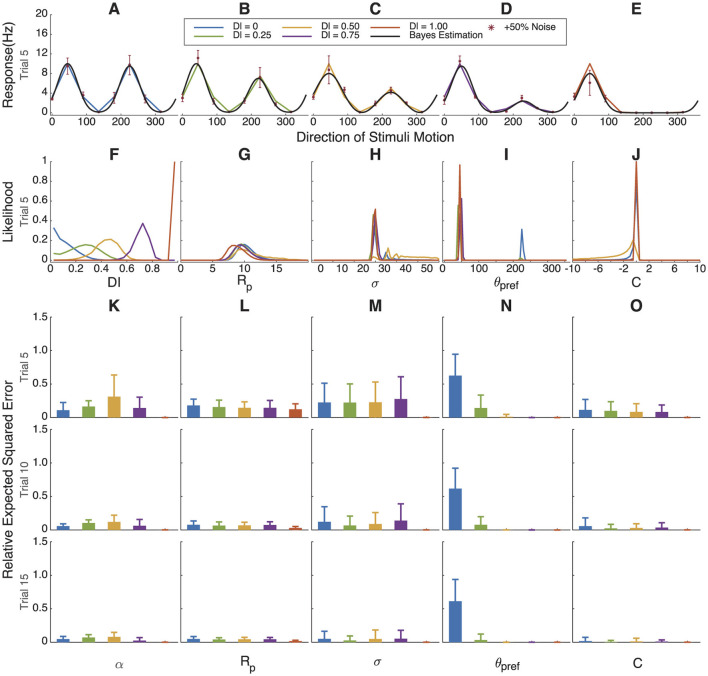
Bayesian estimation results for simulated cells with gradually increasing direction index values. **(A–E)** Simulated response curve, sampling points with +50% Gaussian noise and fitting results. **(F–J)** The marginal likelihoods distribution of *DI*, *R*_*pref*_, σ, θ_*pref*_ and *C*. **(K–O)** Relative error in parameter estimation compared to assuming a uniform distribution for each parameter. Because orientation selectivity is high in all cases, we have certainty about the values except for θ_*pref*_. When direction selectivity approaches 0, then we have uncertainty as to the true angle preference θ_*pref*_.

In Figure 4 we simulated cells with increasing orientation selectivity index values; in Figure 5, we simulated cells with increasing direction selectivity index values. In Figure 4F, the likelihood of the underlying OI value tracks the true value, and θ_*pref*_ (Figure 5I) becomes more certain as the underlying orientation index increases. Similarly, in Figure 4F, the likelihood of the underlying direction index tracks the true value, and again θ_*pref*_ is more certain as the direction selectivity increases.

The relative error between the distribution found by the Bayesian estimation procedure and the true value of the parameters that were used in the simulation is shown in Figures 4K–O, 5K–O. As expected, parameters *R*_*p*_ and *C* can be well estimated regardless of the orientation index, but the tuning width σ, angle preference θ_*pref*_, and α are very uncertain when the orientation index is low. Similarly, when orientation selectivity is high and direction selectivity varies, most parameters can be well estimated except that the preferred angle θ_*pref*_ becomes highly uncertain when direction selectivity is very low.

Another way to quantify Bayesian parameter estimation is to ask how often the true value of the parameter distribution sits within the central *x*% of posterior distribution. One would expect that this would be true approximately *x*% of the time; in other words, that 50% of the time, the true value would lie within the central 50% of the posterior. In Supplementary Figures 1, 2, we show how often the true parameter values in these simulations fall within the central 25th, 50th, and 75th percentiles of the posterior distributions. When the parameter can be well estimated (high orientation or direction selectivity), this is true, and performance falls slightly when the parameter cannot be well estimated.

### 3.5 Impact of sampling angle quantity on Bayesian estimation fitting results

**Figure 6:**
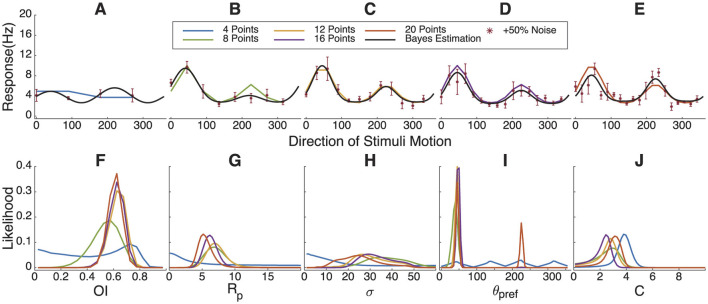
Impact of varying sampling angle quantities on Bayesian estimation results. **(A–E)** Simulated response curve, sampling points with +50% Gaussian noise and fitting results. **(F–J)** The marginal likelihoods distribution of *OI*, *R*_*pref*_, σ, θ_*pref*_ and *C*. In order to estimate the tuning parameters well, one should sample at least 8 directions with visual stimulation.

Experimental time is extremely valuable, and it is important to know how many angle steps might be necessary to estimate the parameters to a desired precision. Each color in the Figure 6 corresponds to a different number of sampling angles, with the legend indicating the number of angles used for each Bayesian estimation fitting. The parameters of the simulated tuning curve are set as (*C* = 2.5, *R*_*pref*_ = 7.5, *R*_*null*_ = 3.75, $\theta_{pref}=45^{•}$, and σ = 30°).

From the likelihood distribution of OI in Figure 6F, it can be observed that four sampling angles produce a poor parameter estimation. There is also a significant likelihood of obtaining a fit with low orientation selectivity due to the accidental collection of angles where the response frequency in the “real” tuning curve is lower. Starting from eight sampling angles, a likelihood peak can be seen around 0.6, although it is somewhat broad. Similarly, in Figures 6G, J, higher sampling angles concentrate the probability peaks at specific values, and the fitting curves align well with the “real” tuning curve. This indicates that eight or more sampling angles meet a minimum requirement for Bayesian estimation. However, under this parameter set, the probability distribution for σ remains broad, with an indistinct peak in the range from 20 to 30.

### 3.6 Bayesian estimation fitting results for neuronal response curves in the ferret primary visual cortex

**Figure 7:**
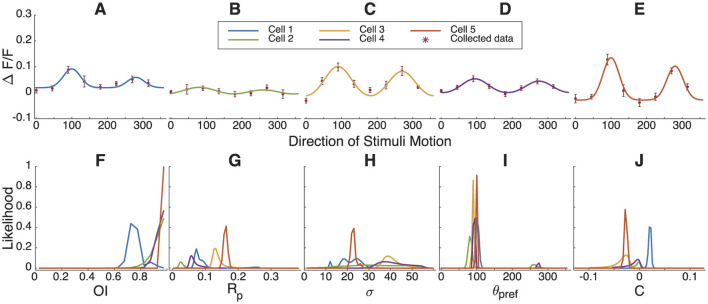
Bayesian parameter estimation of orientation and direction tuning for example cells recorded in ferret visual cortex. **(A–E)** The response of 5 example neurons from the juvenile ferret visual cortex to stimuli moving in different directions and Bayesian parameter estimation results. **(F–J)** The marginal likelihoods distribution of *OI*, *R*_*pref*_, σ, θ_*pref*_ and *C*.

Up to now, we have only examined simulated data for illustration. Next, we analyzed response tuning curves of primary visual cortex neurons from juvenile ferrets using calcium imaging from a prior study (Li et al., 2008). Five example neurons are shown in Figures 7A–E. In Figure 7G, the likelihood distribution of *R*_*pref*_ lies between 0 to 0.2, with the green cell and purple cell showing smaller *R*_*pref*_ values. Nevertheless, all cells exhibit orientation selectivity with high confidence (Figures 7F, H–J), and have narrow ranges of preferred directions, with some cells showing a possibility of preferring the opposite direction (Figure 7I).

### 3.7 Bayesian parameter estimation compared to the bootstrap

Quantifying uncertainty in model parameters when the model is not a member of a well-known class, such as a mean or a generalized linear model, has been challenging. One common method is the bootstrap (Efron and Tibshirani, 1993; Press and Flannery, 1992). Under this procedure, one generates hundreds or thousands of simulated surrogate datasets for each cell by drawing from the original data with replacement. For example, to simulate a surrogate dataset for a cell that was examined with *N* stimulus repetitions of *S* stimuli, one would, for each stimulus *s*, draw *N* response values from the *N* actual response values with replacement, so that some trials are drawn more than once and others drawn not at all. By performing a model fit, such as a least-squares fit, of the hundreds or thousands of surrogate datasets, one can build up a probability distribution of parameter values that reflect the certainty or uncertainty of that parameter.

At first glance, using Bayesian parameter estimation or using the bootstrap for least-squares fits might seem equally appropriate, and that the chief difference might be whether one wanted to perform billions of forward calculations (where the calculations are not iterative and amendable to GPU methods) or thousands of least-squares fits (which are iterative and are less amendable to GPU methods). But we will argue that Bayesian parameter estimation is preferred because of the practical problems with least-squares fitting, including local minima and a frequent need to place constraints on parameters to aid with convergence (Mazurek et al., 2014).

We reanalyzed data from Li et al. (2008). In that work, the authors examined direction tuning in neurons of young ferrets before and after 3-6 hours of exposure to moving stimuli. In the naive state, cells generally exhibited relatively weak tuning for direction, whereas, after motion exposure, cells exhibited stronger tuning. One question raised in that study was whether or not individual cells were likely to have had an eventual direction preference that (a) emerged from an unselective state, (b) was solidified from an initial bias toward that direction preference, or (c) was the result of a reversal of an initial preferred direction preference.

**Figure 8:**
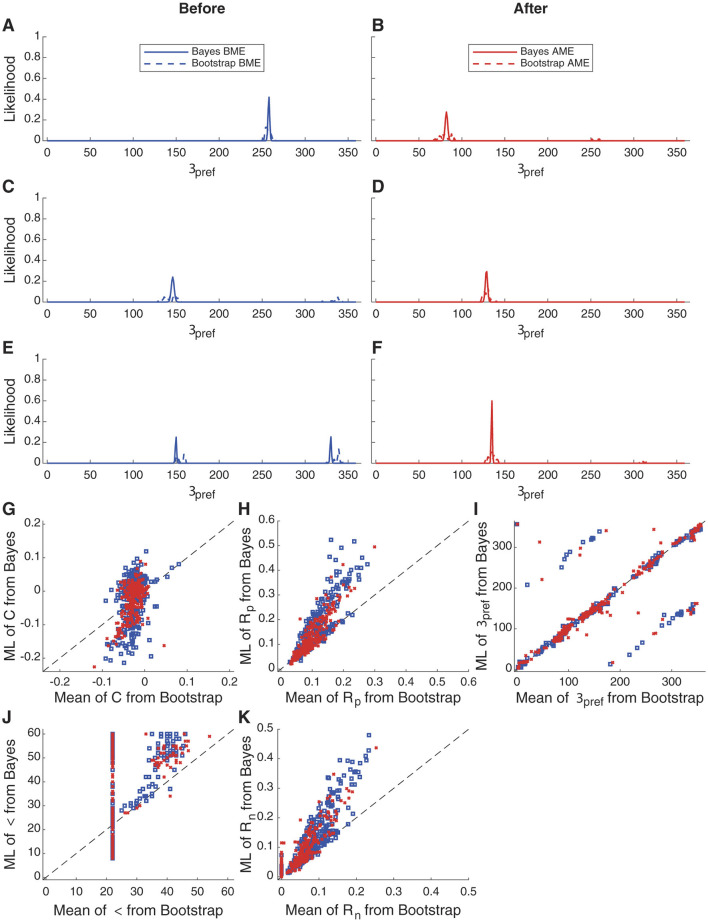
Bayesian estimation and bootstrap fitting results before and after motion exposure. **(A–F)** Probability distribution fitting results for the parameter θ_*pref*_ in three representative cells before and after motion exposure. Results from measurements made before motion exposure are shown in blue while results from measurements made after motion exposure are shown in red. **(G–K)** Fitting results of individual parameters across 262 cells using different models. Each panel shows the relationship between the Bootstrap mean (x-axis) and the Bayesian maximum likelihood (ML) estimate (y-axis) for various model parameters, including *OI*, *R*_*pref*_, σ, θ_*pref*_ and *C*. In general, the results are proportional and the results for θ_*pref*_ are either identical or are occasionally shifted by 180°. However, results for parameter σ are greatly impacted by the floor of the fitting procedure employed with the bootstrap (see Mazurek et al., 2014 for the necessity of this constraint), whereas the Bayesian parameter estimation has no such issue.

Figures 8A–F displays the Bayesian likelihood distributions of θ_*pref*_ for three sample cells before motion exposure and after motion exposure, illustrating changes in tuning properties. In Figures 8A, B, the preferred direction angle shifts 180 degrees, indicating a major and significant reorganization for that cell. Figures 8C, D shows an enhanced response strength at the original preferred angle, suggesting reinforcement of the cell's existing tuning properties. Finally, Figures 8E, F reveals an emergence of a single direction preference, where previously the cell exhibited similar response frequencies in two opposite directions. These findings highlight the effectiveness of Bayesian estimation in capturing nuanced changes in neuronal tuning, similar to bootstrap analysis (Figure 8D).

The advantages of Bayesian parameter estimation become more apparent if we compare bootstrap and Bayesian parameter estimation across the population of 262 cells. Parameter *R*_*p*_ shows a monotonic relationship, indicating that both procedures do a similar job of identifying that parameter, and θ_*pref*_ is the same or, in a few cells that are presumably not very direction-selective, 180° apart. But, in order to converge to a meaningful fit, parameter σ was set at a floor of 22.5° because the angle step in this experiment was 45°. In many bootstrap least-squared fits (Figure 8J), this floor value was the best-fit value. However, in the Bayesian approach, we find that the MLE parameter value spans a broader range. Further, the bootstrap approach often returned the constrained minimal value for *R*_*n*_, which was 0, in the bootstrap approach, whereas the MLE for the Bayesian parameter estimation spanned a wider range(Figure 8K).

Therefore, the Bayesian parameter estimation approach, which dutifully examines the likelihood of different combinations of parameters over many ranges, is immune from the local minima and constraint problems that can plague least-squares fitting. In Bayesian fitting, one has the burden of choosing an appropriate discretization of the parameter space and providing computational resources to carry out the computation, but with modern GPU methods these are not burdensome for relatively simple models.

### 3.8 Test of correctness on a different orientation model

**Figure 9:**
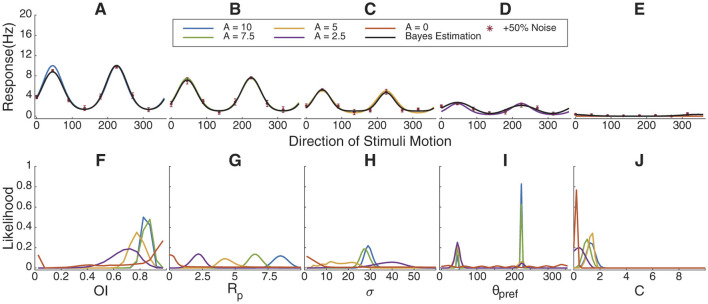
Bayesian parameter estimation on von Mises tuning curves. **(A–E)** Simulated tuning curves from a von Mises function with maximum firing rate (*A*) varied from 10 to 0 in steps of 2.5 sp/s. Data points are tuning curves disturbed by 50% noise and black lines are most-likely values of parameters determined by Bayesian parameter estimation. **(F)** Orientation index (OI) estimations show high values until *A* = 0. **G** Likelihoods for *Rp* in the Bayesian estimation closely match values of *A*. **(H)** σ. **(I)** θ_*pref*_ matches the orientation modulo 180°. **(J)** Estimates for C.

Up to now, we have demonstrated our approach on tuning curves generated from the same function as the response model we are fitting. One may ask if it also does a good job on data that might be generated by similar but not identical processes. To examine the Bayesian parameter estimation performance under these conditions, we generated tuning curves from a von Mises process (Equation 14) while varying the preferred response from 0 to 10 spikes/sec and using a constant amount of noise of 2 spikes/sec across the tuning curve. Results are shown in Figure 9. When *A* is high, θ_*pref*_ and *R*_*p*_ are well estimated by the approach. As *R*_*p*_ drops to 0, the estimate of θ_*pref*_ deteriorates as expected, indicating our uncertainty in determining the true θ_*pref*_ from the data.

### 3.9 Noise model

The Bayesian approach requires that one specify the amount of trial-to-trial noise that is expected in the model. As described in the methods, we started from the prior observations that in cortical neurons the variance or standard deviation of the response across trials is proportional to the mean of the response across trials Softky and Koch (1993) and Shadlen and Newsome (1998) on a log-log plot. We extended this slightly to include a constant term: $\sigma_{noise}\left(m\right)=C_{n}+K*m^{S}$.

**Figure 10:**
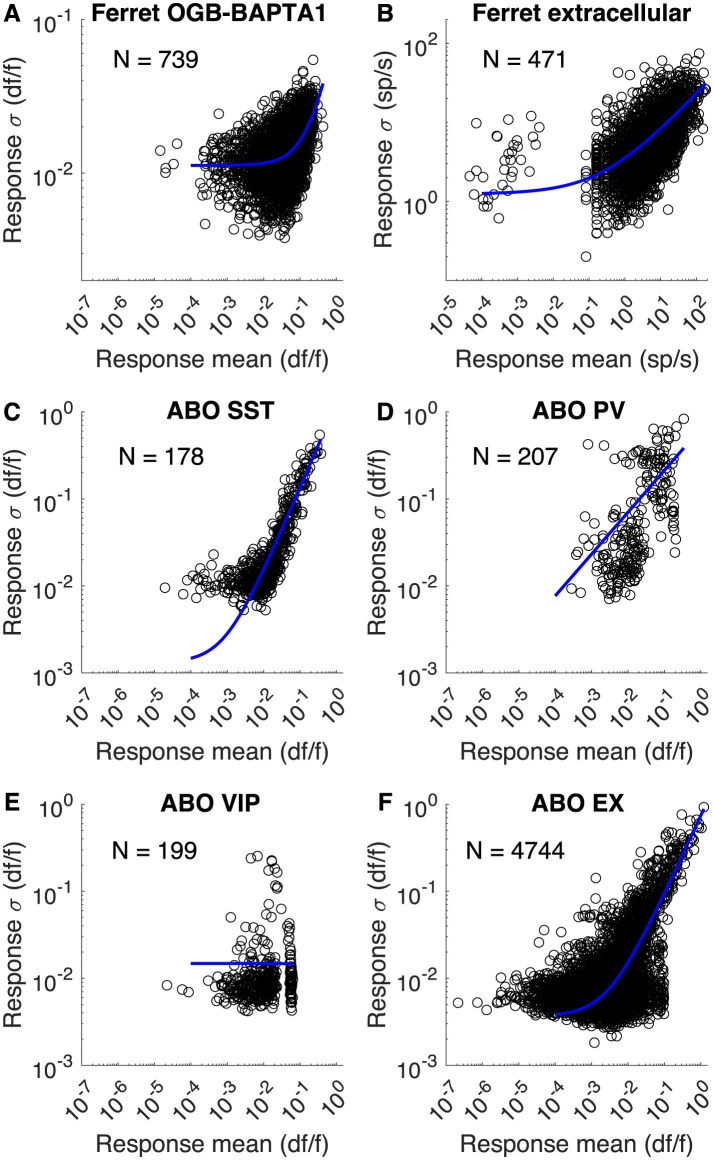
Noise models for different recordings. **(A)** Trial-by-trial response means and standard deviations for 739 ferret neurons labeled with Oregon Green BAPTA-1 AM (Li et al., 2008). 8 responses from each neuron are plotted (different stimulus directions). The best-fit model was *C*_*n*_ = 0.011, *K* = 0.0715, *S* = 1.14. **(B)** Unpublished recordings from Van Hooser lab showing mean and standard deviations of responses to visual stimuli for 471 neurons from ferret visual cortex. *C*_*n*_ = 1.24, *K* = 2.31, *S* = 0.492 **(C–F)** 2-photon calcium imaging responses from different mouse lines expressing gCamp6f and reported in the Allen Brain Observatory (de Vries et al., 2020). Cell numbers as indicated. For SST: *C*_*n*_ = 0.00131, *K* = 1.26, *S* = 0.972. For PV: *C*_*n*_ = 1.9*e*−8, *K* = 0.643, *S* = 0.481. For VIP: *C*_*n*_ = 0.0148, *K* = −5.61, *S* = 3.38. For EX: *C*_*n*_ = 0.00364, *K* = 0.738, *S* = 0.901.

We found that this function characterized the relationship between mean response and noise for a wide range of recordings, including 2-photon calcium imaging using Oregon Green BAPTA-1 AM from the previous section (Li et al., 2008), shown in Figure 10A and extracellular records from ferret (Van Hooser, unpublished observations) (Figure 10B). To further explore how this noise model might suite different applications, we examined several cells from the Allen Brain Observatory's 2-photon dataset (de Vries et al., 2020), where somatostatin-positive (SST) interneurons (Figure 10C), vasoactive intestinal peptide-positive (VIP) interneurons (Figure 10E), parvalbumin-positive (PV) interneurons (Figure 10D), and pan-excitatory cells (Ex) (Figure 10F) were studied in different gCamp6 mouse lines. All cell types were well described by the equation (Figures 8C–F), but, interestingly, VIP interneurons exhibited noise that was largely independent of the mean response rate such that the *K* and *S* parameters were unnecessary. Nevertheless, the strategy of fitting Equation 7 to obtain noise parameters would succeed for all of these cell types.

## 4 Discussion

We derived a Bayesian estimation approach for orientation and direction tuning for neurons or other processes. Least-squares fitting and vector approaches do a good job of identifying model parameters when there is some substantial selectivity, but do not readily produce a measure of certainty or uncertainty when tuning is poor (Mazurek et al., 2014). Bayesian parameter estimation explicitly calculates the likelihood of different parameter values. Until recently, the billions of calculations needed to estimate these parameter distributions were an impediment to the broad use of Bayesian methods, but the new availability of fast GPUs that can perform trillions of floating-point calculations per second makes this feasible even for large populations of neurons.

### 4.1 Modifications of least-squares models

In order to apply Bayesian methods to tuning curves, it may be necessary to modify the original least-squares tuning model, as we did here. The traditional form of the double Gaussian equation (Mazurek et al., 2014) uses independent parameters for the preferred and null responses, and, for least-squares fitting, one can just take the larger of the two to be the preferred response parameter and the smaller to be the null parameter. However, in the Bayesian approach, it is necessary to specify the preferred angle, and we modified the null parameter to be an α times the preferred.

### 4.2 Selecting what to save

Another critical choice in full Bayes parameter estimation is choosing what information to save. Saving the results of billions of computations to disk is not feasible, but one can save smaller descriptions. Here, we saved the most likely parameters and the marginal probabilities of each parameter, but also saved a histogram of several index parameters that were calculated on the full joint distribution. These included the orientation and direction selectivity index values and the circular variance in both orientation and direction space. If one needed the full joint distribution for any reason, it can be recomputed quickly for a single tuning curve, while the marginal probability and index histograms can be saved in a database for aggregate analysis across cells.

### 4.3 Uncertainty and an alternative to the bootstrap

When least-squares methods dominated computation, a common technique to understand the uncertainty of each parameter was the bootstrap (Press and Flannery, 1992). The analyst would create hundreds or thousands of surrogate datasets by drawing individual observations from the originals with replacement, and perform the least-squares fitting on the surrogate datasets. In this way, the analyst would have a distribution of hundreds or thousands of potential values of the fit parameters, and could analyze the uncertainty. Bayesian parameter estimation performs a very similar function, in that one obtains a probability distribution for all parameters, and one can assess uncertainty of all parameters.

This raises the question as to which method might be preferred, Bayesian parameter estimation or least-squares fitting with bootstrap? The downside to Bayesian parameter estimation is a need to specify the values over which to perform the estimation. The resolution of the answer will be exactly that specified. With least-squares fitting, the best-fitting parameters are found to high resolution (often to many significant digits). However, least-squares fits often require constraints in order to make sure the results have physical meaning. In the case of orientation/direction fitting, we usually place a constraint on the tuning width: it can be no less than 1/2 of the angle steps used for visual stimulation (e.g., if the visual stimulus was stepped in 30° steps, then σ must be at least 15°). The results of Figure 8 indicate that this is exactly what happened in our prior analysis, and the Bayesian parameter estimation approach would be preferred. Further, if there were some stereotypy in the fitting procedure that favors some values over others (one might imagine that certain θ_*pref*_ values might emerge for noisy input more often than others if the code were written in a certain way), then this stereotypy will skew the results of the bootstrap approach when the input signal is weak or noisy. Therefore, if the parameters can be specified at a suitable resolution for the problem, we recommend the Bayesian parameter estimation approach instead of the bootstrap.

### 4.4 When it's not worth the effort

The approach we describe here allows one to characterize a variety of properties of orientation and direction tuning, including peak response, preferred angle, and the background activity. If one only wants to assess the degree of orientation or direction tuning, then vector methods such as the circular variance (Mazurek et al., 2014; Ringach et al., 2002) are completely suitable [see simulations in Mazurek et al. (2014)].

### 4.5 Comparison to Bayesian decoding

Bayesian parameter estimation and Bayesian decoding are essentially the same approach that use the same mathematical equations but with slightly different goals. In Bayesian parameter estimation, one is applying Bayesian methods to learn the parameters of a model. In Bayesian decoding, one is often using a model that has already been learned in order to make an inference about some feature of a system, such as the position of an animal given activity in its hippocampal place cells (Brown et al., 1998; Zhang et al., 1998; Karlsson and Frank, 2009; Davidson et al., 2009; Shin et al., 2019). Just as in Bayesian parameter estimation, one can report the “most likely” outcome (for example, the most likely position of an animal) or compute an entire distribution (for example, a distribution of likelihoods that the animal is at any given position in an environment).

### 4.6 Summary

We recommend the Bayesian parameter estimation approach for sensory tuning curves or other response model when it is important to understand the parameter values of the response model and the certainty/uncertainty in these parameters. We recommend this approach above least-squares for finding the most-likely mean values of the parameters, and we recommend this approach above the bootstrap for understanding parameter uncertainty in most cases. We imagine that many studies that seek to characterize the tuning of large numbers of neurons will shortly transition away from least-squares approaches to Bayesian parameter estimation. This article has described a recipe for making the switch.

## Data Availability

The original contributions presented in the study are included in the article/Supplementary material, further inquiries can be directed to the corresponding author. Code and data are available at https://github.com/VH-Lab/vhlab-bayesoridir-matlab
