# Supplementary material for: Bayesian estimation of orientation and direction tuning captures parameter uncertainty
Source: Front Neural Circuits. 2025 Jul 21;19:1542332. doi: 10.3389/fncir.2025.1542332 (PMC12319010; doi:10.3389/fncir.2025.1542332)
Supplement: Supplementary file 2 [file Data_Sheet_2.pdf]

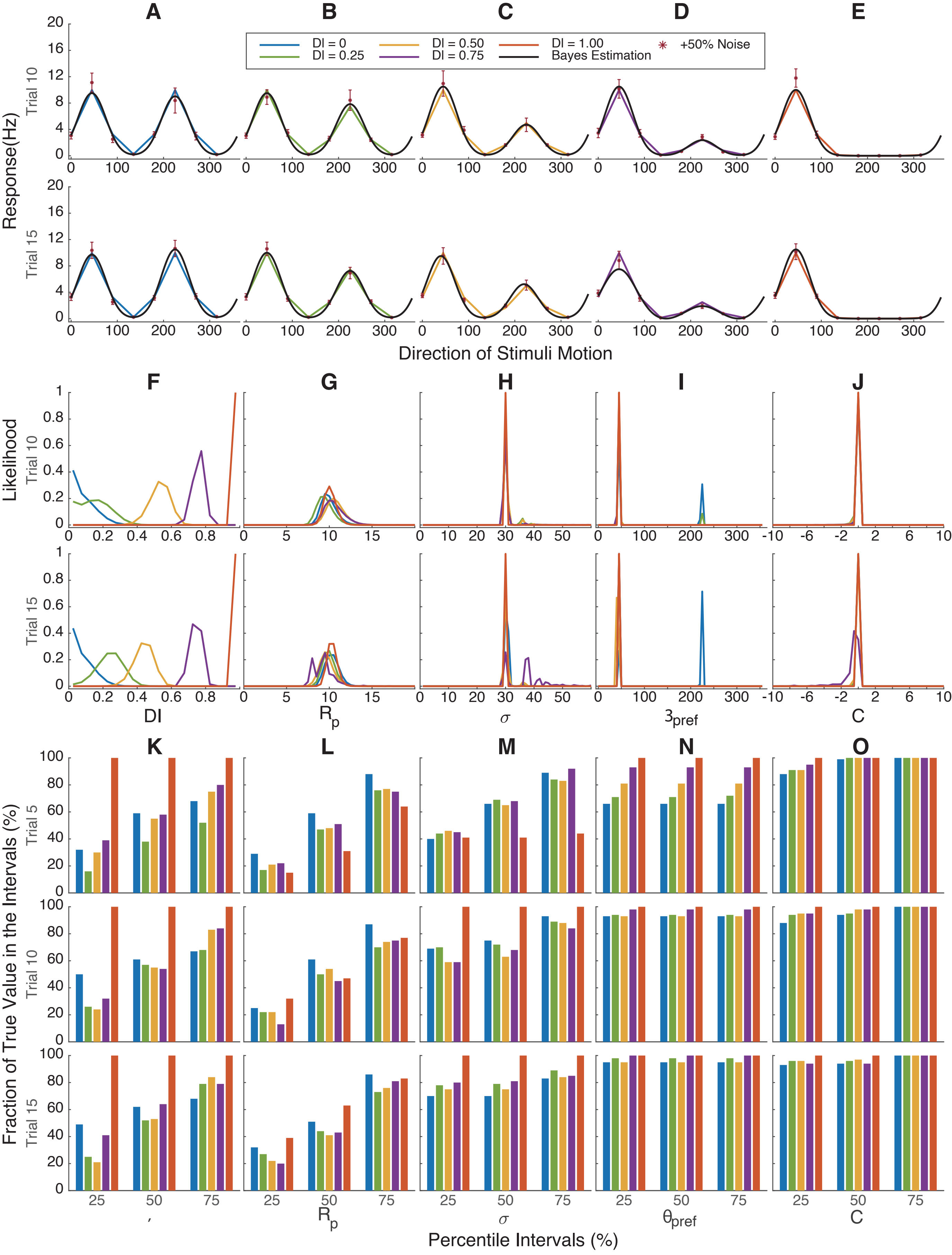

A
B
C
D
E
DI = 0
DI = 0.25
DI = 0.50
DI = 0.75
DI = 1.00
Bayes Estimation
+50% Noise
Response(Hz)
Trial 10
Trial 15
Direction of Stimuli Motion
F
G
H
I
J
Likelihood
DI
R_p
σ
3_pref
C
K
L
M
N
O
Fraction of True Value in the Intervals (%)
Trial 5
Trial 10
Trial 15
25
50
75
R_p
σ
θ_pref
C
Percentile Intervals (%)

Figure S2. Comparing the posterior distribution to the true parameter in direction tuning curve simulations. (A-E) Simulated orientation tuning curves with increasing amounts of underlying direction selectivity; identical to Figure 5A-E. Likelihood distributions returned from single simulations; identical to Figure 5F-J. K-O: Probability that the true value of the parameter is within the 25th, 50th, or 75th percentile of the returned posterior distribution (500 simulations each), and for each of the 5 simulated underlying curves. When signal strength is high, this value matches about 25, 50, or 75% of the time. When signal strength is very low, this value can be lower, depending upon where the true parameter is relative to the range of values explored because we are using a uniform prior. Over time, a non-uniform prior formed on the basis of large datasets could improve this performance.
